# Neural-cadherin expression associated with angiogenesis in non-small-cell lung cancer patients

**DOI:** 10.1038/sj.bjc.6600955

**Published:** 2003-05-27

**Authors:** T Nakashima, C Huang, D Liu, K Kameyama, D Masuya, S Kobayashi, M Kinoshita, H Yokomise

**Affiliations:** 1 Second Department of Surgery, Kagawa Medical University, 1750-1 Miki-cho, Kita-gun, Kagawa 761-0793, Japan; 2 Department of Pathology, Kagawa Medical University, Kagawa, Japan; 3 Gene-Diagnostic Center, Otsuka Assay Laboratory, Otsuka Pharmaceutical Co., Ltd., Tokushima, Japan

**Keywords:** N-cadherin, E-cadherin, angiogenesis, immunohistochemistry, lung cancer

## Abstract

An immunohistochemical analysis for E(epithelial)-cadherin and N(neural)-cadherin expression in relation to tumour angiogenesis was performed in 150 patients with nonsmall cell lung cancer (NSCLC). In all, 71 carcinomas (47.3%) were E-cadherin-negative. Epithelial-cadherin-negative tumours had lymph node metastases significantly more frequently than E-cadherin-positive tumours (*P*=0.0100). On the other hand, 46 carcinomas (30.7%) were N-cadherin-positive. Regarding tumour vascularity, there was no significant correlation between E-cadherin expression and tumour vascular. In contrast, the frequency of hypervascular tumours was significantly higher for N-cadherin-positive carcinomas than for N-cadherin-negative carcinomas (*P*=0.0373). Regarding prognosis, the 5-year survival rate of patients with E-cadherin-negative NSCLCs was significantly lower than that of patients with E-cadherin-positive NSCLCs (*P*=0.0146). In contrast, of the patients with large cell carcinomas, the 5-year survival rate of patients with N-cadherin-positive tumours was significantly lower than that of patients with N-cadherin-negative tumours (*P*=0.0013). A multivariate analysis demonstrated that E-cadherin status (*P*=0.0339) and tumour vascularity (*P*=0.0295) were significant indicators for survival. In conclusion, E-cadherin expression and tumour vascularity are significant prognostic factors of NSCLC patients. Furthermore, N-cadherin expression is associated with tumour angiogenesis, and its expression is one of prognostic factors of patients with large cell carcinomas. Thus, N-cadherin also might play a specific role in undifferentiated large cell carcinomas.

It is widely accepted that malignant tumours are caused by the accumulation of genetic alterations, which could reflect the biological behaviour of tumours (Cordon-Cardo, 1995). In particular, metastasis is specific for malignant tumours, and the control of tumour metastasis is one of the most important problems in the design of therapies for cancer patients. Its initial step is the escape of cells from the primary tumours, which is considered to be dependent on the status of various adhesion molecules, including cadherins, integrins, selectins, and the immunoglobulin superfamily (Pignatelli and Vessey, 1994).

Cadherins are transmembrane glycoproteins that function for calcium-dependent homophilic cell–cell adhesion (Takeichi, 1991). They include several subclasses, such as E (epithelial)-cadherin, N (neural)-cadherin, P (placental)-cadherin and so on. Among these cadherins, E-cadherin is widely expressed in normal epithelial tissues and is linked to the actin cytoskeleton by the catenins (Aberle *et al*, 1996). Experimental studies have demonstrated that E-cadherin acts as a metastatic suppressor gene (Frixen *et al*, 1991; Mbalaviele *et al*, 1996). Previous clinical studies also have revealed that the functional disruption of E-cadherin is associated with tumour dedifferentiation and metastasis in various human cancers including nonsmall cell lung cancers (NSCLCs) (Shibanuma *et al*, 1998; Sulzer *et al*, 1998; Liu *et al*, 2001).

On the other hand, N-cadherin is expressed mainly in the nervous system and in mesenchymal cells, such as myocytes and fibroblasts (Hatta *et al*, 1987). And its expression is associated with a variety of morphogenic events and angiogenesis during development (Hatta and Takeichi, 1986; Blaschuk and Rowlands, 2000). Thus, the function of N-cadherin is suggested to be different from that of E-cadherin. Furthermore, recent studies have demonstrated that N-cadherin expression in tumour tissues is associated with tumour progression, such as epithelial–mesen-chymal transitions (Birchmeier *et al*, 1996), motility, and metastasis (Nieman *et al*, 1999; Hazan *et al*, 2000; Li *et al*, 2001). In addition, several studies on human cancers have reported the presence of the cadherin switching, from E-cadherin to N-cadherin (Hsu *et al*, 1996; Tomita *et al*, 2000).

However, despite of the accumulation of these results regarding N-cadherin, only a few clinical studies on N-cadherin expression in human cancers were reported (Yanagimoto *et al*, 2001), and its true function in human cancers is still unknown. Therefore, to clarify the function of N-cadherin in NSCLCs, we performed a retrospective clinical study on N-cadherin expression and E-cadherin expression by immunohistochemistry. In addition, because the tumour angiogenesis was also reported to be essential to tumour growth and metastasis (Folkman, 1990, 1995), we evaluated intratumoural microvessel density (IMD) using anti-CD34 monoclonal antibody to study the correlation between N-cadherin expression and tumour angiogenesis (Matsuyama *et al*, 1998).

## MATERIALS AND METHODS

### Clinical characteristics of patients

From January 1993 to February 1999, NSCLC patients who underwent surgery at the Second Department of Kagawa Medical University were studied. Tumour-node-metastasis (TNM) staging designations were made according to the international postsurgical pathological staging system. Since advanced stage lung cancer (stage IV) involves several complicated factors and these primary tumour specimens are difficult to obtain by surgical resection, these patients were excluded from this study. In total, 150 patients with lung cancer up to stage IIIB, which included 86 patients with adenocarcinoma, 50 patients with squamous cell carcinoma, and 14 patients with large cell carcinoma which is defined as undifferentiated NSCLC without the component of squamous cell carcinoma, adenocarcinoma (Travis *et al*, 1999), were investigated. The patients' clinical records and histopathological diagnoses were fully documented. This report includes follow-up data as of 10 September 2002. The median follow-up period for all patients was 41.1 months.

### Immunohistochemical staining of E-cadherin, N-cadherin, and CD34

We used a mouse polyclonal antibody for E-cadherin (Clone HECD-1, Takara, Otsu, Japan) diluted at 1 : 400, a mouse monoclonal antibody for N-cadherin (clone 32, BD Bioscience, Franklin Lakes, NJ, USA) diluted at 1 : 1000, and a mouse monoclonal antibody for CD34 (NU-4A1, Nichirei Corporation, Tokyo, Japan) diluted at 1 : 10.

**Figure 1:**
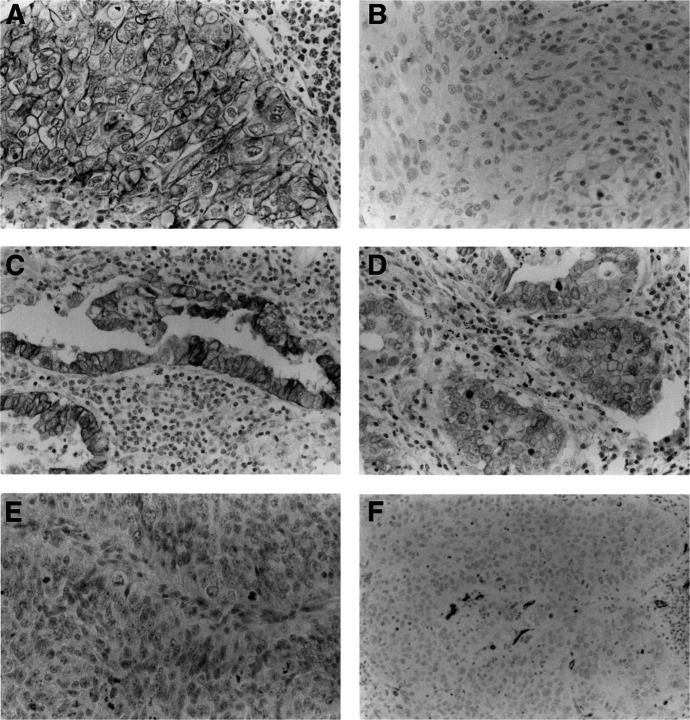
Immunohistochemical staining of human NSCLC tissues using the avidin–biotin–peroxidase complex procedure (original magnification, × 100). (**A**) An E-cadherin-positive carcinoma. (**B**) An E-cadherin-negative carcinoma. (**C**), (**D**) N-cadherin-positive carcinomas. (**E**) A N-cadherin-negative carcinoma. (**F**) Intratumoural microvessel detected by anti-CD34 antibody in a carcinoma.

Formalin-fixed paraffin-embedded tissue specimens were cut into 4-*μ*m sections and mounted on poly-L-lysine-coated slides. The sections were deparaffinised and rehydrated. The slides were then heated in a microwave for 10 min in a 10-*μ*mol l^−1^ citrate buffer solution at pH 6.0, and cooled to room temperature for 20 min. After quenching the endogenous peroxidase activity with 0.3% H_2_O_2_ (in absolute methanol) for 30 min, the sections were blocked for 2 h at room temperature with 5% bovine serum albumin. Subsequently, duplicate sections were incubated overnight with the primary specific antibodies for E-cadherin, N-cadherin, and CD34, respectively. The slides were then incubated for 1 h with biotinylated anti-mouse IgG (Vector Laboratories Inc., Burlingame, CA, USA). The sections were incubated with the avidin–biotin–peroxidase complex (Vector Laboratories Inc.) for 1 h, and the antibody binding was visualised with 3,3′-diaminobenzidine tetrahydrochloride. Finally, the sections were counterstained with Mayer's haematoxylin (Figure 1

). Normal bronchus epithelium and normal mucosal glands within the tumour sections were used as positive internal controls for staining of E-cadherin. Sections of resected lung tumours known to express N-cadherin were used as positive controls for staining of N-cadherin. Sections incubated with normal mouse IgG served as negative reaction controls for staining of E-cadherin and N-cadherin.

All of the immunostained sections were reviewed by two pathologists who had no knowledge of the patients' clinical status. Patients with contradictory scores were reevaluated jointly on a second occasion and consensus was reached. For the evaluation of immunostaining of E-cadherin and N-cadherin, in cases with multiple areas of low intensity, five areas selected at random were scored; in sections where all of the staining appeared intense, one random field was selected. At least 200 tumour cells were scored per × 40 field. When ⩾50% of the tumour cells in a given specimen were positively stained for E-cadherin, the sample was classified as E-cadherin-positive. When <50% of the cells were stained, it was classified as E-cadherin-negative. In addition, when ⩾20% of the tumour cells in a given specimen were positively stained for N-cadherin, the sample was classified as N-cadherin-positive. When <20% of the cells were stained, it was classified as N-cadherin-negative.

For microvessel quantification, the three most highly vascularised areas detected by CD34 immunostaining were initially selected under the × 40 field, and a × 200 field (0.785 mm^2^ per field) was used to count vessels in each of these areas (Figure 1F). Vessels of a calibre larger than approximately eight red blood cells and vessels with thick muscular walls were excluded from the count. Single endothelial cells or clusters of endothelial cells, with or without lumen, were considered to be individual vessels. Finally, the average of three × 200 field counts was recorded as the IMD. Tumours with IMD ⩾90 were classified as hypervascular, while tumours with IMD <90 were classified as hypovascular.

### Statistical analysis

The overall cancer-specific survival was defined from the date of the operation to the date of cancer-related death. The statistical differences in E-cadherin and N-cadherin expression in relation to several other clinical and pathological parameters including IMD were assessed by the *χ*^2^ test and the *t*-test. The Kaplan–Meier method was used to estimate the probability of overall survival as function of time, and differences in the survival of subgroups of patients were compared with Mantel's log-rank test. Cox's proportional-hazards regression model was used to study the effects of different variables on survival. All *P*-values were based on two-tailed statistical analysis and a *P*-value <0.05 was considered to indicate statistical significance.

## RESULTS

### Epithelial-cadherin expression in NSCLCs

Normal bronchus epithelium and normal mucosal glands had positive membranous expression of E-cadherin. Intratumoural E-cadherin staining also appeared in the form of a membranous staining pattern, with or without cytoplasmic staining (Figure 1A, B). Of the 150 tumours studied, 79 carcinomas (52.7%) were E-cadherin-positive, and 71 carcinomas (47.3%) were E-cadherin-negative (Table 1

**Table 1:** Distribution of 150 NSCLC patients according to E-cadherin, N-cadherin status, and tumour vascularity

		E-cadherin			**N-cadherin**			**Tumour vascularity**		
**Variables**	***n***	**Positive**	**Negative**	***P*-value**	**Positive**	**Negative**	***P*-value**	**Hypervascular**	**Hypovascular**	***P*-value**
*Tumour status*										
T1	62	36	26	0.1629	20	42	0.9245	39	23	0.0540
T2	50	27	23		16	34		20	30	
T3	7	1	6		2	5		3	4	
T4	31	15	16		8	23		20	11	
*Nodal status*										
N0	106	63	43	0.0100	35	71	0.3265	58	48	0.9847
N1, N2, N3	44	16	28		11	33		24	20	
*Pathological stage*										
Stage I	86	53	33	0.0378	28	58	0.3198	45	41	0.6656
Stage II	15	4	11		3	12		8	7	
Stage IIIA	18	7	11		8	10		9	9	
Stage IIIB	31	15	16		7	24		20	11	
*Differentiation*										
Well	54	32	22	0.2815	17	37	0.9859	37	17	0.0375
Moderately	50	27	23		15	35		23	27	
Poorly	46	20	26		14	32		22	24	
*Histology*										
Adenocarcinoma	86	50	36	0.1088	31	55	0.1344	58	28	0.0070
Squamous cell carcinoma	50	25	25		10	40		17	33	
Large cell carcinoma	14	4	10		5	9		7	7	
Total number of patients	150	79	71		46	104		82	68	

). Of the 86 adenocarcinomas, 36 tumours (41.9%) were E-cadherin-negative. Of the 50 squamous cell carcinomas, 25 tumours (50.0%) were E-cadherin-negative. Of the 14 large cell carcinomas, 10 tumours (71.4%) were E-cadherin-negative. The frequency of E-cadherin-negative tumours was likely to be greater in large cell carcinoma than any other tumour histology. Furthermore, E-cadherin-negative tumours had lymph node metastases significantly more frequently than E-cadherin-positive tumours (39.4 *vs* 20.3%, *P*=0.0100) (Table 1). In addition, advanced stage tumours were significantly more frequent in E-cadherin-negative tumours than in E-cadherin-positive tumours (53.5 *vs* 32.9%, *P*=0.0378).

### Neural-cadherin expression in NSCLCs

Intratumoural N-cadherin staining also appeared in the form of a membranous staining pattern, with or without cytoplasmic staining. Of the 150 tumours, 46 carcinomas (30.7%) were N-cadherin-positive, and 104 carcinomas (69.3%) were N-cadherin-negative (Figure 1C–E and Table 1). Of the 86 adenocarcinomas, 31 tumours (36.0%) were N-cadherin-positive. Of the 50 squamous cell carcinomas, 10 tumours (20.0%) were N-cadherin-positive. Of the 14 large cell carcinomas, five tumours (35.7%) were N-cadherin-positive. There was no significant relation between N-cadherin expression status and tumour histology. In addition, there was no significant relation between N-cadherin expression status and other patient prognostic factors, such as tumour status, nodal status, pathological stage, and tumour differentiation (Table 1).

### Relation between E-cadherin expression and N-cadherin expression

With respect to 150 NSCLCs, 28 tumours (35.4%) were N-cadherin-positive among the 79 E-cadherin-positive carcinomas, while 18 tumours (25.4%) were N-cadherin-positive among the 71 E-cadherin-negative carcinomas. There was no significant relation between N-cadherin expression and E-cadherin expression in NSCLCs. However, regarding 14 undifferentiated large cell carcinomas, only one tumour (25.0%) was N-cadherin-positive among four E-cadherin-positive tumours, while four tumours (40.0%) were N-cadherin-positive among 10 E-cadherin-negative tumours.

### Tumour vascularity in NSCLCs

The IMD in the 150 NSCLCs ranged from 24.0. to 394.7, with a mean of 110.6±65.3. In all, 82 carcinomas (54.7%) were hypervascular, and 68 carcinomas (45.3%) were hypovascular (Table 1). Regarding tumour histology, of the 86 adenocarcinomas, 58 tumours (67.4%) were hypervascular. Of the 50 squamous cell carcinomas, 17 tumours (34.0%) were hypervascular. Of the 14 large cell carcinomas, seven tumours (50.0%) was hypervascular. The frequency of hypervascular tumours in adenocarcinomas was significantly greater than that in squamous cell carcinomas (*P*=0.0007). However, there were no significant correlation between IMD and other prognostic factors, such as tumour status, nodal status, tumour differentiation.

### Relation between cadherin expressions and tumour vascularity

With respect to E-cadherin status, of the 79 E-cadherin-positive carcinomas, 46 tumours (58.3%) were hypervascular; of the 71 E-cadherin-negative carcinomas, 36 tumours (50.7%) were hypervascular. There was no significant correlation between E-cadherin expression and tumour vascularity.

**Figure 2:**
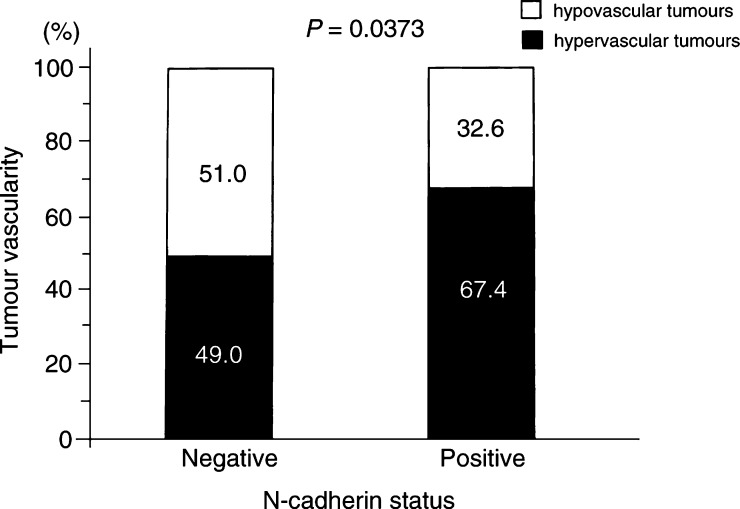
Tumour vascularity in relation to N-cadherin status in NSCLCs.

Regarding N-cadherin status, of the 46 N-cadherin-positive carcinomas, 31 tumours (67.4%) were hypervascular; of the 104 N-cadherin-negative carcinomas, 51 tumours (49.0%) were hypervascular. The frequency of hypervascular tumours was significantly higher for N-cadherin-positive carcinomas than for N-cadherin-negative carcinomas (*P*=0.0373; Figure 2

). With respect to tumour histology, of the 86 adenocarcinomas, the frequencies of hypervascular tumours were 74.2% in N-cadherin-positive carcinomas, 63.6% in N-cadherin-negative carcinomas. Of the 50 squamous cell carcinomas, the frequencies of hypervascular tumours were 50.0% in N-cadherin-positive carcinomas, 30.0% in N-cadherin-negative carcinomas. Of the 14 large cell carcinomas, the frequencies of hypervascular tumours were 60.0% in N-cadherin-positive carcinomas, 44.4% in N-cadherin-negative carcinomas.

### Overall survival of NSCLC patients in relation to E-cadherin status, N-cadherin status, and tumour vascularity

The 5-year survival rates of 150 NSCLC patients according to E-cadherin status, N-cadherin status and tumour vascularity are shown in Table 2

**Table 2:** Five-year survival rate of 150 NSCLC patients according to E-cadherin, N-cadherin status, and tumour vascularity

	E-cadherin			**N-cadherin**			**Tumour vascularity**		
**Variables**	**Positive**	**Negative**	***P*-value**	**Positive**	**Negative**	***P*-value**	**Hypervascular**	**Hypovascular**	***P*-value**
*Tumour status*									
T1	79.1	59.8	0.0328	76.8	68.5	0.7681	70	72.4	0.6472
T2	59.9	45.5	0.4792	45	57.2	0.512	40	62.5	0.0106
T3	0.0	25	0.7741	0	33.3	0.0389	0	50	0.4547
T4	40.4	24.1	0.2268	60	22	0.0909	31.8	37.9	0.9706
*Nodal status*									
N0	73.2	58.4	0.1863	71.3	65.7	0.5415	61.0	76.2	0.0613
N1,N2,N3	31.1	25.2	0.2680	24.2	27.7	0.6718	26.3	26.5	0.2061
*Pathological stage*									
Stage I	78.4	65.6	0.3910	74.9	72.9	0.6786	70.4	77.8	0.3797
Stage II	50.0	40.0	0.6728	33.3	45.5	0.4972	25.0	66.7	0.1021
Stage IIIA	21.4	20.0	0.4972	18.8	22.2	0.4935	22.2	16.7	0.2155
Stage IIIB	40.4	24.1	0.2268	53.6	25.9	0.2244	31.8	37.9	0.9706
*Differentiation*									
Well	80.7	57.1	0.0284	87.5	63.7	0.1619	68.6	75.0	0.4029
Moderately	62.2	47.1	0.3806	27.6	64.2	0.1245	41.1	67.3	0.0285
Poorly	34.8	32.8	0.4907	53.3	33.4	0.4948	33.3	44.9	0.1870
*Histology*									
Adenocarcinoma	64.3	46.8	0.0777	68.5	51.1	0.1518	57.3	57.1	0.8660
Squamous cell carcinoma	68.3	50.4	0.1619	60.0	58.0	0.4981	44.1	68.3	0.0349
Large cell carcinoma	50.0	30.0	0.4934	0.0	55.6	0.0013	14.3	57.1	0.0535
Total	64.4	45.4	0.0146	59.6	54.0	0.6596	50.8	62.2	0.0378

**Figure 3:**
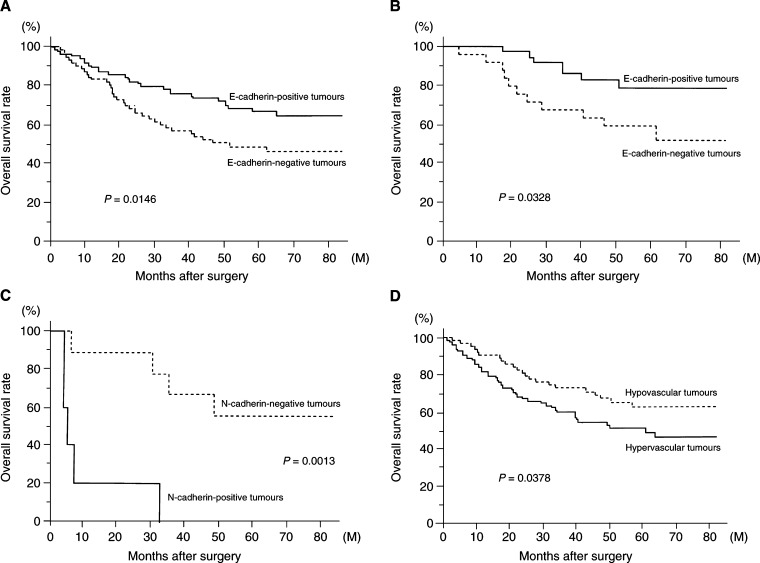
(**A**) Overall survival of 150 patients with NSCLC in relation to their E-cadherin status. (**B**) Overall survival of 62 patients with T1 NSCLC in relation to their E-cadherin status. (**C**) Overall survival of 14 patients with large cell carcinoma in relation to their N-cadherin status. (**D**) Overall survival of 150 patients with NSCLC in relation to their tumour vascularity.

. With respect to E-cadherin status, the 5-year survival rate of patients with E-cadherin-negative NSCLCs was significantly lower than that of patients with E-cadherin-positive NSCLCs (45.4 *vs* 64.4%, *P*=0.0146, Figure 3A

). Especially, the 5-year survival rate of patients with E-cadherin-negative T1 tumours was significantly lower than that of patients with E-cadherin-positive T1 tumours (79.1 *vs* 59.8%, *P*=0.0328, Figure 3B).

In contrast, there was no significant difference in the 5-year survival rate between patients with N-cadherin-positive NSCLCs and patients with N-cadherin-negative NSCLCs (59.6 *vs* 54.0%). However, of the 14 patients with undifferentiated large cell carcinomas, the 5-year survival rate of patients with N-cadherin-positive tumours was significantly lower than that of patients with N-cadherin-negative tumours (0.0 *vs* 55.6%, *P*=0.0013, Figure 3C).

With respect to tumour vascularity, the 5-year survival rate of patients with hypervascular NSCLCs was significantly lower than that of patients with hypovascular NSCLCs (50.8 *vs* 62.2%, *P*=0.0378, Figure 3D). Regarding tumour histology, the 5-year survival rate of patients with hypervascular squamous cell carcinomas was significantly lower than that of patients with hypovascular squamous cell carcinomas (44.1 *vs* 68.3%, *P*=0.0349).

A multivariate analysis using Cox proportional-hazards regression model was performed to evaluate prognostic factors for NSCLC patients, as shown in Table 3

**Table 3:** Multivariate regression analysis in predicting survival of 150 NSCLC patients

**Histology**		**Nonsmall cell lung cancer**		**Adenocarcinoma**		**Squamous cell carcinoma**		**Large cell carcinoma**	
**Variables**	**Assigned score**	**Hazard ratio**	***P*-value**	**Hazard ratio**	***P*-value**	**Hazard ratio**	***P*-value**	**Hazard ratio**	***P*-value**
*E-cadherin*									
Positive	0	1.736	0.0339	1.389	0.3503	2.662	0.0912	0.111	0.2586
Negative	1								
*Tumour vascularity*									
Hypovascular	0	1.785	0.0295	1.121	0.7693	5.529	0.0053	24.815	0.0312
Hypervascular	1								
*Tumour status*									
T1	1	1.399	0.0013	1.352	0.0194	1.756	0.0816	7.020	0.0374
T2	2								
T3	3								
T4	4								
*Nodal status*									
N0	0	1.829	<0.0001	1.719	0.0007	2.916	0.0162	15.526	0.0160
N1, N2, N3	1								
*Smoking*									
Nonsmoker	0	1.485	0.1860	1.534	0.2838	2.461	0.3222	23.088	0.0834
Smoker	1								
*Age* (year)									
<60	0	0.898	0.7386	0.681	0.3459	2.034	0.5356	19.941	0.0530
⩾60	1								

. Epithelial-cadherin status (hazard ratio=1.736, *P*=0.0339) and tumour vascularity (hazard ratio=1.785, *P*=0.0295) were significant indicators for survival of NSCLC patients. With respect to tumour histology, tumour vascularity was also a significant indicator for survival of patients with squamous cell carcinomas (hazard ratio=5.529, *P*=0.0053), and patients with large cell carcinomas (hazard ratio=24.815, *P*=0.0312).

## DISCUSSION

The cadherins are members of a large family of transmembrane glycoproteins that mediate calcium dependent, homophilic cell–cell adhesion and play an important role in the maintenance of normal tissue architecture (Takeichi, 1990). Among this family, E-cadherin is widely expressed in normal epithelial cells. Numerous studies have demonstrated the importance of the E-cadherin/catenin complex in maintaining the normal phenotype of epithelial cells (Frixen *et al*, 1991; Mbalaviele *et al*, 1996). Although the actual mechanisms responsible for its functional disruption in human cancers are still not fully clarified (Blaschuk *et al*, 1995; Nawrocki *et al*, 1998), their dysfunction causes dedifferentiation and invasive tumours. In a previous experimental study, invasive fibroblastic-like carcinoma cells could be converted to a noninvasive phenotype by transfection of E-cadherin cDNA (Frixen *et al*, 1991). Previous clinical studies in human cancers, including NSCLCs, also have demonstrated that dysfunction of E-cadherin/catenin complex is associated with dedifferentiation (Bohm *et al*, 1994), lymph node metastasis (Sulzer *et al*, 1998), and a poor prognosis (Sulzer *et al*, 1998). The present study has also revealed that decreased expression of E-cadherin is associated with lymph node metastasis and a poor prognosis in NSCLC patients. Thus, E-cadherin acts as a metastatic suppressor gene in human NSCLCs.

Consistent with these results, E-cadherin promotes tight cell–cell adhesion in a zipper-like fashion, restricting cell movement. On the other hand, recent experimental studies have demonstrated that N-cadherin could promote a dynamic adhesion state. Neural-cadherin is responsible for the interaction between endothelial cells and other surrounding cell types expressing N-cadherin such as vascular smooth muscle cells and pericytes (Navarro *et al*, 1998). This kind of adhesion of N-cadherin was suggested to play important roles in various biological behaviours, including morphogenesis (Takeichi, 1991) and angiogenesis (Gerhardt *et al*, 1999, 2000) during development and epithelial–mesenchymal transition during tumour progression (Islam *et al*, 1996; Tran *et al*, 1999).

With respect to tumour progression, N-cadherin expression could mediate an epithelial–mesenchymal transition and angiogenesis. At first, the epithelial–mesenchymal transition is associated with typical for some carcinoma cells late in tumour progression and correlated with metastatic potential (Birchmeier *et al*, 1996). In an experimental study, transfection of antisense N-cadherin into a cancer cell line with a scattered fibroblastic phenotype resulted in reversion to a normal-appearing squamous epithelial cell, interestingly, with increased E-cadherin expression (Islam *et al*, 1996). In addition, transfection of N-cadherin into a normal-appearing squamous epithelial cell line resulted in a scattered fibroblastic phenotype, with downregulation of E-cadherin. Another study also revealed that a N-cadherin-positive carcinoma cell line displayed an invasive potential after intraperitoneal injection into SCID mice (Tran *et al*, 1999).

Recently, basic studies have shown the presence of the interactions between N-cadherin and fibroblast growth factor (FGF) receptor (Doherty and Walsh, 1996; Saffell *et al*, 1997). Hazan *et al*. (2000) demonstrated that treatment with FGF-2 induced N-cadherin-expressing breast carcinoma cells into a more invasive phenotype, with upregulation of matrix metalloproteinase MMP-9. Other experimental studies have also shown that transfection of N-cadherin can promote motility in human cancer cell lines (Nieman *et al*, 1999; Li *et al*, 2001).

On the other hand, N-cadherin expression was reported to mediate angiogenesis during development. N-cadherin-expressing cells can interact with N-cadherin-expressing vascular endothelial cells during development (Gerhardt *et al*, 1999, 2000). Then, the interaction of N-cadherin and FGF receptor promotes the FGF-2 signal pathway, which was reported to induce vascular endothelial growth factor expression (Seghezzi *et al*, 1998). Therefore, N-cadherin-expressing tumour cells might induce angiogenesis through the interaction with N-cadherin-expressing vascular endothelial cells during tumour progression. In addition, the tumour angiogenesis is also essential to tumour growth and metastasis (Folkman, 1990, 1995).

Despite of the accumulation of these results regarding N-cadherin, only a few clinical studies on N-cadherin expression in human cancers were reported (Soler *et al*, 1997; Yanagimoto *et al*, 2001). Therefore, we performed the present clinical study on the expression of N-cadherin and E-cadherin in NSCLC patients.

At first, our present study revealed that N-cadherin expression in NSCLCs is significantly associated with tumour vascularity. This is the first clinical report demonstrating a correlation between N-cadherin expression and angiogenesis in human cancers. Especially, the frequencies of hypervascular tumours in N-cadherin-expressing tumours were likely to be higher than that in N-cadherin-negative tumours, among squamous cell carcinoma (50.0 *vs* 30.0%), and among large cell carcinoma (60.0 *vs* 44.4%). In addition, of the large cell carcinomas, the frequency of N-cadherin-positive tumours was likely to be greater in E-cadherin-negative tumours than in E-cadherin-positive tumours (40.0 *vs* 25.0%). Furthermore, among the large cell carcinomas, survival of patients with N-cadherin-positive tumours was significantly lower than that of patients with N-cadherin-negative tumours. However, there was no significant correlation between N-cadherin status and lymph node metastasis.

These results might suggest that in some populations of NSCLCs, from well-differentiated squamous cell carcinomas to undifferentiated large cell carcinomas, inactivation of E-cadherin is an early event in the tumour progression, and activation of the inappropriate cadherin, such as N-cadherin, would be a subsequent event, which could promote angiogenesis and poor survival. This concept of cadherin switching (Cavallaro *et al*, 2002) has been reported in some kinds of human cancers, such as melanoma (Hsu *et al*, 1996) and prostate cancer (Tomita *et al*, 2000). In the present study, however, there was no significant inverse correlation between E-cadherin expression and N-cadherin. This might be partly because undifferentiated carcinoma is relatively rare among NSCLCs.

On the other hand, there was no such a relation regarding N-cadherin expression in adenocarcinomas. Thus, different cellular mechanisms might be responsible in the progression of squamous cell carcinomas and adenocarcinomas (Bohm *et al*, 1994). In the present study, although tumour vascularity was a significant prognostic factor both in patients with squamous cell carcinoma and in patients with large cell carcinoma, there was no significant difference in the survival of patients with adenocarcinoma in relation to tumour vascularity. This finding might be partly because most of adenocarcinomas were hypervascular. In addition, reduced expression of integrin, another kind of adhesion molecules, is associated with the prognosis of patients with adenocarcinoma of the lung (Adachi *et al*, 1998).

In conclusion, the present study of NSCLC patients has demonstrated that E-cadherin expression and tumour vascularity are significant prognostic factors of NSCLC patients. Furthermore, N-cadherin expression is associated with tumour angiogenesis, and its expression is one of the prognostic factors of patients with undifferentiated large cell carcinomas. Thus, N-cadherin also might play a specific role in NSCLCs, especially in undifferentiated large cell carcinomas.
